# Response to Drs. Igor Burstyn and George Luta’s letter: Advice on better utilization of validation data to adjust odds ratios for differential exposure misclassification (recall bias)

**DOI:** 10.5271/sjweh.4232

**Published:** 2025-07-01

**Authors:** Henrik Albert Kolstad, Jesper Medom Vestergaard, MIT,, Jens Peter Bonde, Sadie Costello, Annett Dalbøge, Åse Marie Hansen, Ann Dyreborg Larsen, Anne Helene Garde

**Affiliations:** 1 Occupational and Environmental Medicine, Danish Ramazzini Centre, Aarhus University Hospital, Aarhus, Denmark.; 2 Department of Clinical Medicine, Aarhus University, Denmark.; 3 Department of Occupational and Environmental Medicine, Bispebjerg and Frederiksberg Hospital, Denmark.; 4 Environmental Health Science, School of Public Health, University of California, Berkeley, USA.; 5 The National Research Centre for the Working Environment, Denmark.; 6 Department of Public Health, University of Copenhagen, Denmark.

We want to thank Drs. Burstyn & Luta (1) for their recognition of our recent study (2) suggesting that estimates of breast cancer risk following retrospective self-reported night shift work are inflated by recall bias. The main strength of the study was a gold standard based on individual, prospective, objective and detailed information on night shift work that allowed validation of self-reported night shift work obtained after breast cancer was diagnosed – the usual situation for case–control studies (3). The study confirmed what textbooks have long taught but rarely documented empirically (4–6).

We also want to thank Drs. Burstyn & Luta for their advice on how we could have utilized this precious dataset not only for simple but also probabilistic and Bayesian quantitative bias analyses. Even if highly instructive, this may still require strong statistical involvement.

Using data provided in our paper, Drs. Burstyn & Luta’s bias-corrected odds ratio (OR) estimate of breast cancer following night shift work was centered around 1.0 (95% credible interval 0.3–1.7) and suggests that recall bias could completely, and not only partly as in our analysis (OR 1.05; correctly computed 95% confidence interval 0.88–1.27), explain the observed associations between night shift work and breast cancer found in case–control studies with retrospective self-reported exposure information. This finding strengthens our concern that breast cancer studies based on retrospective self-reports of night shifts may not provide convincing evidence.

The gold standard of this validation study was based on a cohort of healthcare workers with day-by-day night shift information from a pay roll register and has earlier been used for breast cancer risk assessment showing no increased risk (7). A recent Swedish study using comparable data neither showed an overall increased risk (8). However, these studies included only information on recent night shift work. The next step should be a follow up of the cohorts when information on more distant night shift work becomes available with an emphasis on analyses that explore the timing of shift work on breast cancer risk.
